# Prevalence and spectrum of fungal pathogens in pulmonary tuberculosis patients

**DOI:** 10.6026/973206300221179

**Published:** 2026-02-28

**Authors:** Priyanka Prasad, Suneel Kumar Ahirwar, Anju Mahor, Deepak Bansal, Manish Purohit

**Affiliations:** 1 Department of Microbiology, MGM, Medical College Indore, Madhya Pradesh, India; 2 Department of Respiratory Medicine, MGM Medical College, Indore, Madhya Pradesh, India

**Keywords:** Pulmonary tuberculosis (PTB), fungal co-infection, *aspergillus*, *Candida*, prevalence

## Abstract

Pulmonary fungal infections are increasingly recognized as significant co-infections in patients with pulmonary tuberculosis (PTB). Therefore, it is of interest to determine the prevalence and spectrum of fungal pathogens in patients with suspected and confirmed PTB. A cross-sectional study was conducted on 180 patients at MGM Medical College, Indore, India, with sputum samples processed for fungal culture. Fungal culture positivity was observed in 20% of the patients, with *aspergillus* species being the most commonly isolated pathogen. Thus, we show the need for routine mycological examination in PTB patients to facilitate early diagnosis and appropriate treatment.

## Background:

Pulmonary tuberculosis (PTB) is a major public health concern worldwide, particularly in low- and middle-income nations. India bears a significant share of the worldwide tuberculosis burden, accounting for roughly one-fourth of all reported cases each year [1]. Although effective antitubercular therapy has considerably improved patient outcomes, many PTB patients experience long-term pulmonary complications such as fibrotic alterations, bronchiectasis, and cavitary lesions [2]. These structural defects, together with altered local immune responses, promote colonization and infection by opportunistic fungal infections. Pulmonary fungal infections are increasingly recognized as significant co-infections in patients with active or previously treated tuberculosis, particularly in TB-endemic areas [3]. Pulmonary fungal infections frequently have clinical and radiological symptoms similar to tuberculosis, such as chronic cough, hemoptysis, fever, weight loss and cavitary lung lesions [4]. As a result, fungal infections may go unnoticed or misdiagnosed as persistent or recurring tuberculosis, resulting in ineffective treatment, delayed antifungal therapy, and increased morbidity. Because of their capacity to colonize pre-existing pulmonary cavities and damaged lung parenchyma, *Aspergillus* species are the most commonly implicated in post-tubercular lung illness of any fungal pathogen [5]. Chronic pulmonary aspergillosis and other *Aspergillus*-related diseases are increasingly being described in individuals with tuberculosis. *Candida* species, while widely found as respiratory tract commensals can also function as opportunistic pathogens in patients with weak immunity or underlying lung illness [6]. Other filamentous fungi have been recorded less frequently, although they may add to the overall incidence of pulmonary mycoses. Several studies from various regions of India have revealed fungal isolation rates ranging from 14% to 30% among patients with pulmonary tuberculosis, emphasizing the clinical significance of fungal co-infection in this group [7]. However, the prevalence and scope of fungal diseases differ greatly depending on geographic location, environmental exposure, patient characteristics, and laboratory testing procedures [8]. Data on the spectrum of pulmonary fungal infections among patients with suspected and confirmed pulmonary tuberculosis from Central India are scarce. Given the similar clinical symptoms of tuberculosis and pulmonary fungal infections, as well as the potential influence of ndiagnosed fungal disease on patient therapy, it is critical to establish the local incidence and spectrum of fungal pathogens [9]. Therefore, it is of interest to report the local incidence and spectrum of fungal pathogens in this patient population to better understand the clinical implications and guide appropriate management strategies.

## Materials and Methods:

A cross-sectional observational study was conducted in the Department of Microbiology in collaboration with the Department of Respiratory Medicine at MGM Medical College, Indore, India. A total of 180 patients with suspected or microbiologically confirmed pulmonary tuberculosis attending outpatient and inpatient services were included in the study.

## Inclusion criteria:

[1] Patients clinically suspected of pulmonary tuberculosis

[2] Microbiologically confirmed pulmonary tuberculosis patients

[3] Patients willing to provide informed consent

## Exclusion criteria:

[1] Patients already receiving antifungal therapy

[2] Patients unwilling to participate in the study

[3] Inadequate, contaminated or insufficient sputum samples

## Sample collection:

Early morning sputum samples were collected in sterile, wide-mouthed, leak-proof containers after instructing patients regarding proper oral hygiene. Samples were transported promptly to the microbiology laboratory for further processing.

## Laboratory processing:

## Direct microscopy:

All sputum samples were examined using 10-20% potassium hydroxide (KOH) mount for the detection of fungal elements such as hyphae or yeast cells.

## Fungal culture:

Samples were inoculated onto Sabouraud dextrose agar (SDA) with and without antibiotics and incubated at 25°C and 37°C. Cultures were observed periodically for up to four weeks for evidence of fungal growth.

## Identification of fungal isolates: 

Fungal isolates were identified based on colony morphology, growth characteristics, and microscopic features using lactophenol cotton blue (LPCB) mount. Yeast isolates were further identified using germ tube test, CHROM agar, and Dalmau plate method, wherever applicable.

## Data collection:

Relevant demographic and clinical details, including age, sex, tuberculosis status (suspected or confirmed), and laboratory findings, were recorded using a predesigned proforma.

## Statistical analysis:

Data were entered and analyzed using standard statistical software. The prevalence of fungal culture positivity was expressed as frequencies and percentages. The spectrum of fungal pathogens isolated from sputum samples was analyzed using descriptive statistics. Categorical variables were summarized as proportions. Wherever applicable, associations between categorical variables were assessed using the chi-square test. A p-value of <0.05 was considered statistically significant.

## Results:

A total of 180 patients with suspected or microbiologically confirmed pulmonary tuberculosis were included in the study (1). Among patients with microbiologically confirmed pulmonary tuberculosis, *Aspergillus* *fumigatus* remained the predominant isolate (25%), followed by *Aspergillus* flavus and *Candida* albicans (18.8% each). *Candida* glabrata constituted 12.5% of isolates in confirmed cases, while other fungal species were isolated less frequently (Table 4). Analysis of fungal isolates showed a predominance of *Aspergillus* and *Candida* species among the culture-positive samples. *Aspergillus* *fumigatus* was the most frequently isolated species, accounting for 22.2% of isolates, followed by *Candida* albicans (19.4%) and *Aspergillus* niger (13.9%). Other isolates included *Aspergillus* flavus, *Candida* tropicalis, and *Candida* glabrata. Less commonly isolated fungi included *Aspergillus* terreus, *Candida* krusei, *Fusarium* spp., and *Exerohilum spp* Table 3). Direct microscopic examination using KOH mount demonstrated fungal elements in 37 samples. Fungal culture positivity was observed in 36 (20%) patients. Correlation between KOH mount and fungal culture findings revealed that 27 samples were positive by both methods, while 9 samples were culture positive but KOH negative. Conversely, 10 samples were KOH positive but culture negative. The remaining 134 samples were negative by both KOH mount and culture (Table 2). The demographic distribution showed a predominance of males (108, 60%) compared to females (72, 40%). The majority of patients belonged to the 21-40 years age group (41.1%), followed by the 41-60 years age group (36.6%) (Table 1).

## Discussion:

Pulmonary fungal infections are increasingly being recognized as important co-infections among patients with suspected and confirmed pulmonary tuberculosis, especially in tuberculosis-endemic regions. In the present study, fungal culture positivity was observed in 20% of cases, highlighting that a considerable proportion of patients evaluated for pulmonary tuberculosis may harbor concomitant fungal infections. Similar prevalence rates have been reported in previous Indian studies, where fungal isolation among pulmonary tuberculosis patients ranged between 15% and 30% [10]. Variations in prevalence across studies may be attributed to differences in geographic location, environmental exposure, patient population, immune status, and diagnostic methodologies employed. In the present study, Aspergillus species were the predominant fungal isolates, with *Aspergillus* *fumigatus* being the most frequently isolated species. This finding is consistent with earlier studies from tuberculosis-endemic regions, which have documented A. *fumigatus* as the most common etiological agent of post-tubercular pulmonary mycoses due to its thermotolerance, airborne nature, and ability to colonize pre-existing lung cavities [11]. Structural lung damage following tuberculosis, including cavitation and fibrosis, provides an ideal niche for Aspergillus colonization and subsequent infection. *Candida* species constituted the second most common group of fungal isolates in this study. Although *Candida* species are often considered colonizers of the respiratory tract, repeated isolation in patients with chronic pulmonary symptoms and underlying lung disease may indicate a pathogenic role rather than mere colonization [12]. Previous studies have emphasized that *Candida* isolation from sputum samples in tuberculosis patients should be interpreted cautiously, but persistent recovery, especially in symptomatic individuals, warrants clinical attention [13]. The isolation of less common filamentous fungi such as *Fusarium spp.* and *Exserohilum* spp., though infrequent, is noteworthy. These fungi are environmental molds and have been increasingly reported as opportunistic pathogens in patients with chronic lung disease and compromised pulmonary defenses [14]. Their presence in the current study underscores the importance of comprehensive fungal identification rather than restricting investigations to common fungal pathogens alone. The correlation between KOH mount and fungal culture findings in the present study highlights the limitations of direct microscopy as a sole diagnostic tool. A number of culture-positive cases were negative on KOH examination, indicating that reliance solely on microscopy may result in under diagnosis of fungal infections. Similar observations have been documented in earlier studies, which recommend fungal culture as an essential component of diagnostic workup for suspected pulmonary mycoses [15]. Overall, the findings of this study reinforce the growing evidence that pulmonary fungal infections are a significant yet under-recognized contributor to respiratory morbidity among patients evaluated for pulmonary tuberculosis. Incorporation of routine mycological investigations in suspected and confirmed pulmonary tuberculosis cases may facilitate early detection of fungal co-infections and improve patient management.

## Conclusion:

Patients with suspected or proven pulmonary tuberculosis frequently experience pulmonary fungal co-infection. The current investigation shows a high prevalence of fungal culture positive, with *Aspergillus* species, particularly *Aspergillus* *fumigatus*, being the most commonly isolated pathogens, followed by *Candida* species. Routine mycological assessment of sputum samples in patients with pulmonary tuberculosis may aid in the early diagnosis of fungal infections, leading to better clinical care and prognosis.

## Figures and Tables

**Figure 1:**
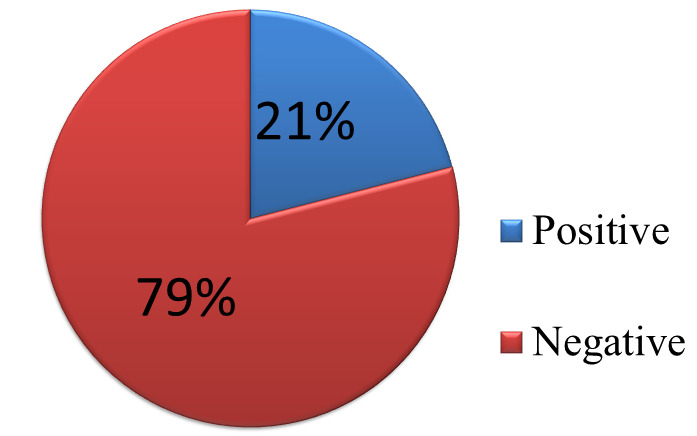
KOH mount positivity among study participants

**Table 1:** Distribution of patients according to age and sex (n = 180)

**AGE GROUP**	**MALE**	**FEMALE**	**Total**
0-20	3	11	14 (7.7%)
21-40	47	27	74 (41.1%)
41-60	39	27	66 (36.6%)
61-80	18	7	25(13.8%)
>80	1	0	1(0.5%)

**Table 2:** Correlation between KOH Mount and fungal culture

**KOH Result**	**Culture Positive**	**Culture Negative**
Positive	27	10
Negative	9	134

**Table 3:** Microbial profile of suspected and confirmed PTB patients

**Fungal Species**	**Number of Isolates**	**Percentage (%)**
*Aspergillus fumigatus*	8	22.20%
*Candidaalbicans*	7	19.40%
*Aspergillus Niger*	5	13.90%
*Aspergillus flavus*	4	11.10%
*Candida tropicalis*	4	11.10%
*Candida glabrata*	3	8.30%
*Aspergillus terreus*	2	5.60%
*Candida krusei*	1	2.80%
Fusarium spp.	1	2.80%
*Exerohilum spp.*	1	2.80%

**Table 4:** Spectrum of fungal isolates in confirmed cases of PTB patients

**Fungal species**	**Number of Isolates**	**Percentage (%)**
*Aspergillus fumigatus*	4	25%
*Aspergillus flavus*	3	18.80%
*Candida albicans*	3	18.80%
*Candida glabrata*	2	12.50%
*Aspergillus niger*	1	6.20%
*Candida tropicalis*	1	6.20%
*Candida krusei*	1	6.20%
*Exerohilum*	1	6.20%
